# Health-Related Quality of Life and Psychosocial Outcomes in Patients With Type 2 Diabetes Mellitus: A Bibliometric Analysis

**DOI:** 10.1155/jdr/5164503

**Published:** 2025-08-28

**Authors:** Guojun Liu, Tianjiao Li, Chaofan Chen, Ningkun Xiao

**Affiliations:** ^1^Inner Mongolia Key Laboratory of Life Health and Bioinformatics, School of Life Science and Technology, Inner Mongolia University of Science and Technology, Baotou, China; ^2^Institution of Natural Sciences and Mathematics, Ural Federal University, Yekaterinburg, Russia; ^3^Department of Physical Education, Tsinghua University, Beijing, China; ^4^Department of Immunochemistry, Institution of Chemical Engineering, Ural Federal University, Yekaterinburg, Russia; ^5^Laboratory for Brain and Neurocognitive Development, Department of Psychology, Institution of Humanities, Ural Federal University, Yekaterinburg, Russia

**Keywords:** bibliometric, health-related quality of life, HRQoL, psychosocial outcomes, quality of life, Type 2 diabetes mellitus, well-being

## Abstract

**Background:** Health-related quality of life (HRQoL) has become a critical focus in managing Type 2 diabetes mellitus (T2DM), emphasizing the need to integrate physiological, psychological, and social dimensions into clinical practice. Despite the growing prevalence of T2DM worldwide, particularly in low- and middle-income countries, the global research landscape of HRQoL remains unevenly distributed.

**Objectives:** This study is aimed at systematically analyzing the global research trends, key contributors, and influencing factors of HRQoL in patients with T2DM using bibliometric methods, providing insights to guide future research and targeted interventions.

**Methods:** A total of 6470 articles published before October 31, 2024, were retrieved from the Web of Science Core Collection (WoSCC). We analyzed publication trends, national and institutional contributions, author impact, journal distribution, and keyword co-occurrence using bibliometric tools. Factors influencing HRQoL were identified through a comprehensive literature review, focusing on physiological, psychological, and social determinants.

**Results:** The number of publications in the field has grown rapidly since 2000, with 40.03% of all articles published in the past 5 years. The United States led global research output with 1620 articles and 98,354 citations, followed by China (622 articles) and the United Kingdom (529 articles). However, China's average citations per article (15.50) lagged significantly behind developed countries such as the United Kingdom (62.70) and the United States (60.70). Key factors influencing HRQoL included psychological health (e.g., depression and anxiety), sleep disturbances, socioeconomic status, lifestyle, and diabetes-related complications. Early research focused on metabolic control and complications, whereas recent studies have increasingly emphasized integrating psychosocial factors and patient-centered management strategies.

**Conclusions:** HRQoL research in T2DM reflects a shift toward holistic, patient-centered approaches that address the complex interplay of clinical, psychological, and social factors. Future research should prioritize addressing disparities in low- and middle-income countries, leveraging international collaboration, and utilizing advanced digital health technologies to enhance HRQoL monitoring and interventions. These efforts will be critical in improving the overall well-being and long-term outcomes of patients with T2DM globally.

## 1. Introduction

Type 2 diabetes mellitus (T2DM) is a chronic, multisystem metabolic disorder that poses a major global public health challenge [1]. It accounts for over 90% of diabetes cases worldwide and is a leading contributor to the growing burden of disease, disability, and mortality [2, 3]. The development of T2DM results from a complex interaction of genetic susceptibility, lifestyle factors such as unhealthy diet and physical inactivity, obesity, and aging [4]. These factors collectively lead to insulin resistance and impaired insulin secretion, causing persistent hyperglycemia [2, 4].

T2DM profoundly impacts patients' overall health by significantly increasing their risk of developing a range of complications and comorbidities. These include cardiovascular disease [4–6], neuropathy [4, 5], diabetic foot [2], diabetic retinopathy [2, 5], renal failure [2], heart failure [6], depression [6], and sleep disorders [7]. These complications adversely affect patients' ability to learn, work, socialize, and engage in physical activity. They severely diminish quality of life (QoL) across multiple dimensions—including physical health, mental well-being, social relationships, and self-perception—and, in some cases, may even be life-threatening [2]. Therefore, to enable clinicians and caregivers to comprehensively understand the overall health and well-being of patients with T2DM and to develop more targeted and effective clinical management strategies, it is essential to expand traditional clinical metrics—which primarily focus on glycemic control and treatment adherence—to encompass a broader, multidimensional assessment of health status and well-being [7, 8].

The concept of health-related quality of life (HRQoL) has evolved significantly in recent years, encompassing a wide range of dimensions closely linked to health, including physical, psychological, emotional, spiritual, and behavioral, self-perceived health, personal beliefs, and social functioning [2, 9–11]. A growing body of research has demonstrated that HRQoL is a critical measure for evaluating the health status, well-being, and mortality risk of patients with chronic diseases [3, 12]. By focusing on the patient's perspective and emphasizing their perceived health, HRQoL provides valuable insights into the overall impact of disease and its interventions on patients' lives, offering patients, clinicians, and caregivers actionable guidance on treatment decisions, management strategies, and preventive measures.

With the increasing volume of research on HRQoL in patients with T2DM, a systematic analysis of the research hotspots and trends in this field has become crucial. Such an analysis not only helps researchers identify current priorities and key challenges in the field but also facilitates the integration of research findings, promoting academic collaboration and knowledge sharing [11, 13]. Therefore, we conducted a comprehensive bibliometric analysis of HRQoL research in patients with T2DM to deepen the understanding of this field. We hope that this study will draw greater attention from researchers, clinicians, caregivers, and patients to HRQoL in T2DM, enhance recognition of the overall health status of patients, and provide personalized psychosocial support, educational programs, and comprehensive care measures, ultimately improving the HRQoL and overall well-being of patients with T2DM.

## 2. Methods

### 2.1. Literature Search and Data Collection

This study utilized the Web of Science Core Collection (WoSCC)—specifically, the Science Citation Index Expanded (SCIE) and the Social Sciences Citation Index (SSCI)—as the primary data source. WoSCC is globally recognized as an authoritative academic database and is among the most widely used platforms for bibliometric analysis, thereby ensuring the scientific validity and reliability of the study results [12].

The search strategy focused on two core themes: “T2DM” and “HRQoL,” along with their associated synonyms. The specific search query was structured as follows: (TS = (“Diabetes Mellitus, Type 2”) OR TS = (“NIDDM”) OR TS = (“Type 2 Diabetes Mellitus”) OR TS = (“Type 2 Diabetes”) OR TS = (“Diabetes, Type 2”) OR TS = (“Diabetes Mellitus, Adult-Onset”) OR TS = (“Ketosis-Resistant Diabetes Mellitus”) OR TS = (“Diabetes Mellitus, Non Insulin Dependent”) OR TS = (“Diabetes Mellitus, Stable”) OR TS = (“Maturity Onset Diabetes Mellitus”) OR TS = (“MODY”) OR TS = (“Diabetes Mellitus, Slow Onset”) OR TS = (“Maturity Onset Diabetes”)) AND (TS = (“Health Related Quality Of Life”) OR TS = (“Quality of Life”) OR TS = (“Life Quality”) OR TS = (“HRQOL”) OR TS = (“Health-Related Quality Of Life”) OR TS = (“Mental Health”) OR TS = (“Mental Hygiene”) OR TS = (“Psychological Health”) OR TS = (“Psychological Well-being”) OR TS = (“Psychological Well Being”)).

**Figure 1:**
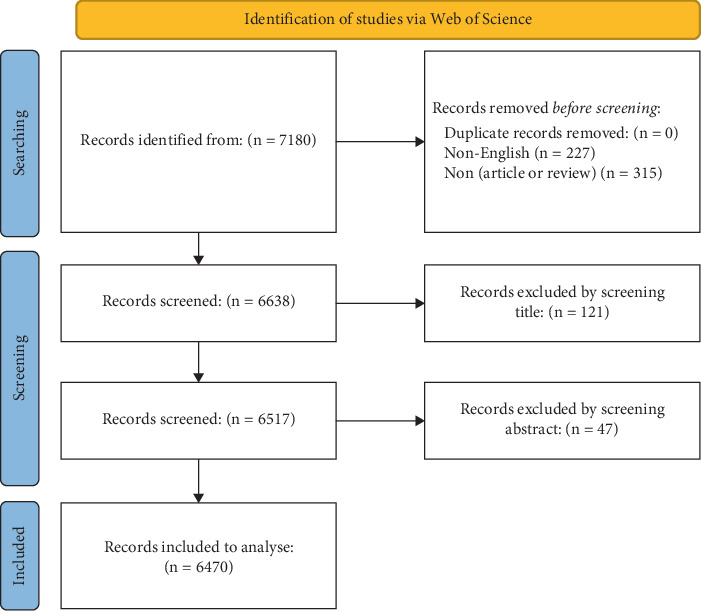
Flow chart of included and excluded studies.

The search was limited to articles published up to October 31, 2024. Only English-language documents classified as “article” or “review” were included. Two researchers independently screened the titles and abstracts of all retrieved records. Duplicates and studies that did not meet the inclusion criteria were excluded. The final dataset was exported in plain text and table-delimited formats, containing key bibliographic and content information including titles, authors, publication years, abstracts, keywords, citation counts, and journals (see Figure 1).

### 2.2. Data Analysis

We imported the exported data into Excel 2019 (Microsoft, Washington, United States) for initial organization and calculated the number of publications and corresponding citation counts. The dataset was then analyzed using the https://bibliometric.com platform and the Bibliometrix package in R software (Version 4.0.3) to explore collaborative relationships among authors, journals, countries, institutions, and keywords.

In the visualization analysis, the size of each node was proportional to its associated parameter weight—such as publication volume or citation frequency—where larger weights corresponded to larger nodes. Each node represented a specific element (e.g., country, institution, author, or keyword), offering a comprehensive visual overview of global research collaboration networks and key thematic hotspots in the field.

## 3. Results

### 3.1. Publication Volume and Growth Trends

**Figure 2:**
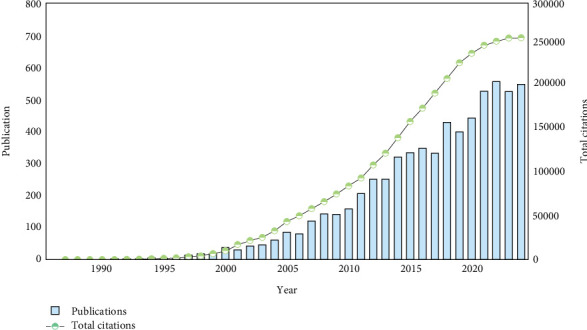
The number of annual publications and citations on HRQoL in patients with T2DM.

As of October 31, 2024, a total of 7180 articles related to HRQoL in patients with T2DM were retrieved from the WoSCC. The final analysis included 6470 articles, after screening and excluding 710 articles that did not meet the inclusion criteria. The analysis revealed distinct changes in publication trends over time. Prior to 2000, the growth in publications was relatively slow. However, since 2000, the number of publications has increased rapidly, with a notable surge in the last 5 years (2020–2024), during which 2590 articles were published, accounting for 40.03% of the total. This trend reflects the growing attention of researchers to the HRQoL of patients with T2DM. Citation data show that the field is changing quickly. The number of citations has been going up steadily since 2002, showing that the field is becoming more important (see Figure 2 for details).

### 3.2. Country/Region and Institutional Analysis

Globally, 120 countries and regions have contributed to research on HRQoL in patients with T2DM. The first article in this field was published in 1987 by Kaplan in the *Journal of General Internal Medicine*, demonstrating that combining diet and exercise significantly improves HRQoL with non–insulin-dependent diabetes mellitus [14].

**Figure 3:**
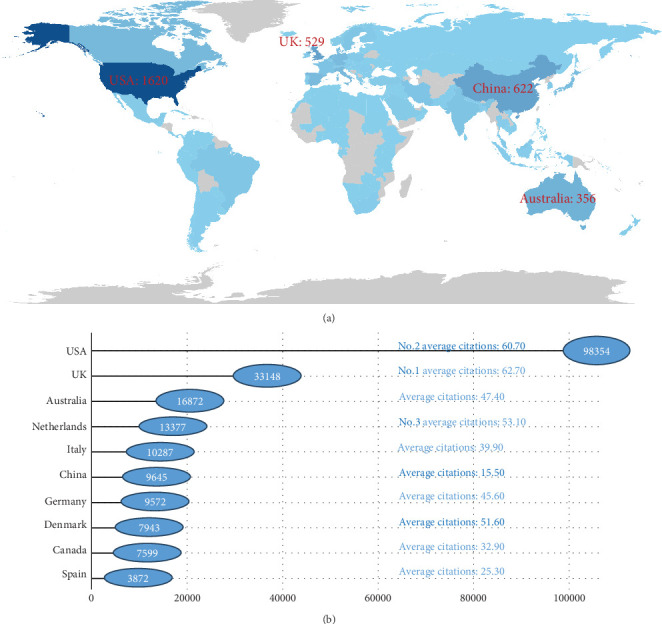
Top 10 countries with the highest publications and citations on HRQoL in patients with T2DM. (a) Top 10 countries with the highest publications on HRQoL in patients with T2DM. (b) Top 10 countries with the highest citations on HRQoL in patients with T2DM.

An analysis of corresponding authors revealed that the United States has a dominant position in this field. As of the retrieval date, the United States had published 1620 articles, accounting for 25.04% of the total, with 1334 articles authored independently and 286 articles coauthored with other countries (see Table 1 and Figure 3 for details). China ranked second with 622 articles (9.61%), of which 504 were authored independently, and 118 were coauthored with other countries. The United Kingdom ranked third with 529 articles (8.18%), followed by Australia (356 articles, 5.50%), Italy (258 articles, 3.99%), the Netherlands (252 articles, 3.89%), Canada (231 articles, 3.57%), Germany (210 articles, 3.25%), Japan (184 articles, 2.84%), and Denmark (154 articles, 2.38%).

From the perspective of collaboration, the United Kingdom demonstrated the highest level of international collaboration, with 41.21% of its articles coauthored with other countries, followed by Australia (38.20%) and Denmark (36.36%). In contrast, Japan exhibited the lowest international collaboration rate, with only 10.87% of its articles involving coauthorship with other countries.

In terms of citations, the United States led with 98,354 citations, followed by the United Kingdom with 33,148 citations. Other countries in the Top 10 included Australia (16,872 citations), the Netherlands (13,377 citations), Italy (10,287 citations), China (9645 citations), Germany (9572 citations), Denmark (7943 citations), Canada (7599 citations), and Spain (3872 citations). Regarding average citations per article, the United Kingdom ranked first (62.70 citations), followed by the United States (60.70 citations) and the Netherlands (53.10 citations). In contrast, China's average citation count was only 15.50, indicating room for improvement in its citation impact (see Figure 3 for details).

**Figure 4:**
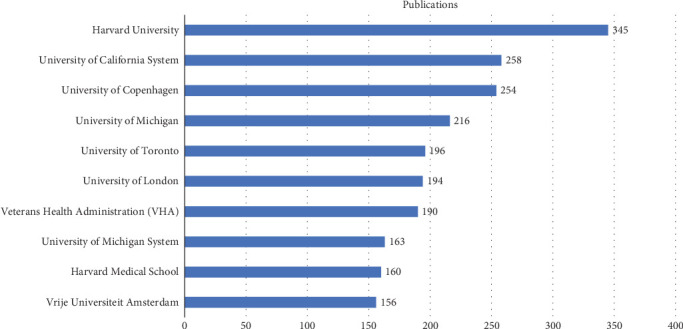
Top 10 institutions with the highest publications on HRQoL in patients with T2DM.

Notably, many of the leading institutions have utilized a variety of HRQoL metrics in their studies, such as the SF-36, EQ-5D, and WHOQOL instruments. Other commonly used HRQoL tools include the SF-12 (a shorter version of SF-36), the Diabetes Quality of Life (DQoL) measure, the Audit of Diabetes-Dependent Quality of Life (ADDQoL), the Health Utilities Index (HUI), and the Patient-Reported Outcomes Measurement Information System (PROMIS) (see Table 2 for details). A closer examination of these widely adopted instruments across institutions may provide valuable insights into how patient-centered outcomes are assessed and compared in the context of Type 2 diabetes research. Institutional analysis revealed that 4805 institutions worldwide contributed to the publication of 6470 articles. Harvard University ranked first with 325 articles, followed by the University of California System (258 articles) and the University of Copenhagen (254 articles). Other institutions in the Top 10 included the University of Michigan (216 articles), the University of Toronto (196 articles), the University of London (194 articles), the Veterans Health Administration (VHA, 190 articles), the University of Michigan System (163 articles), Harvard Medical School (160 articles), and Vrije University Amsterdam (156 articles) (see Figure 4 for details). Among these top institutions, six are based in the United States, while the remaining four are located in Denmark, the United Kingdom, Canada, and the Netherlands—further highlighting the dominant contribution of the United States to this research field.

### 3.3. Author Analysis

In the field of HRQoL research for T2DM, a total of 29,892 authors have contributed to the body of literature. To facilitate researchers' understanding of the key contributors in this field, we summarized the Top 10 authors based on their publication output (see Table 3 for details).

Kamlesh Khunti from the University of Leicester, United Kingdom, is the most prolific author in this field, with 55 publications between 2006 and 2024. His research primarily focuses on diabetes education, self-management, and the impact of physical activity on T2DM [15],, as well as exploring depression prevalence [16], treatment effectiveness, cost-efficiency, and patient satisfaction in the context of HRQoL [17–20]. Frans Pouwer from the University of Southern Denmark has published 40 articles between 1999 and 2024, emphasizing psychological health, well-being, and psychological interventions for patients with T2DM [21–23]. His work also includes examining the impact of hypoglycemia on HRQoL [24]. Another prolific author, Melanie J. Davies, also from the University of Leicester, has collaborated extensively with Kamlesh Khunti and published 39 articles during the same period. Her research spans the effects of physical activity, health interventions, and educational programs on patient outcomes [25] and studies on sleep quality, psychological health, and HRQoL [26, 27].

F. J. Snoek from the Amsterdam Public Health Research Institute, the Netherlands, has published 37 articles between 1994 and 2024. His research explores online platforms and self-help applications in reducing psychological problems and fatigue among patients with T2DM [28], as well as light therapy and psychological interventions for improving symptoms of comorbid depression and well-being [29, 30]. He has also studied the relationship between insulin treatment optimization, glycemic control, and HRQoL [31]. Similarly, Guy E. H. M. Rutten from the University Medical Centre Utrecht has focused on depression symptoms, well-being, and HRQoL [32] and examined the efficacy of self-management education and psychological support in alleviating patient distress [33–35].

In the United States, E. Egede from the University at Buffalo has contributed 30 publications between 2010 and 2024, focusing on the effects of comorbid depression, perceived control, social support, self-care, and social discrimination on HRQoL [10, 36–38]. Additionally, during this time, Ping Zhang from the Centers for Disease Control and Prevention (CDC) has written 30 papers looking into how poor sleep, anxiety, and cognitive impairments affect HRQoL. He has also written about how structured lifestyle interventions like tai chi and exercise can improve HRQoL [39–42]. Kristina Secnik Boye from Eli Lilly and Company has contributed 29 articles, focusing on the effects of tirzepatide on HRQoL and the use of patient-reported outcomes to evaluate the emotional impact of treatment [43–45].

In Italy, Antonio Nicolucci from the Center for Outcomes Research and Clinical Epidemiology has also published 29 articles between 2001 and 2022. His research includes the impact of second-line antidiabetic therapies on HRQoL [19] and studies on diabetes-related distress, economic burden, and patient satisfaction [46, 47]. Finally, Jeffrey A. Johnson from the University of Alberta, Canada, has published 27 articles from 2003 to 2022, focusing on the relationship between physical activity and HRQoL in patients with T2DM [48] and the application of EQ-5D-5L in HRQoL research [49].

Among these Top 10 authors, three are based in the United States, two in the United Kingdom, and two in the Netherlands, with one each from Denmark, Italy, and Canada. F. J. Snoek has the highest local citation count (478), followed by Guy E. H. M. Rutten (339) and Kamlesh Khunti (328). These leading authors' contributions have shaped critical areas of HRQoL research, such as psychological health, lifestyle interventions, and treatment evaluation, laying a strong foundation for further advancements in the field.

### 3.4. Journal Analysis

This study included 6470 articles distributed across 1400 journals. Using Bradford's law, these journals were classified into three groups, each accounting for approximately one-third of the total articles, to identify the core journals in the field [50]. The analysis revealed that Zone 1 contains 29 core journals, Zone 2 includes 174 journals, and Zone 3 covers 1197 journals.

In Zone 1, the Top 10 journals demonstrated a high concentration of publications (see Table 4 for details). *Diabetes Research and Clinical Practice* ranked first, publishing 168 articles, accounting for 2.6% of the total. *Diabetic Medicine* (164 articles) and *Diabetes Care* (149 articles) trailed behind. Other journals in the Top 10 included *BMJ Open* (115 articles), *PLOS One* (102 articles), *Diabetes Therapy* (99 articles), *Cochrane Database of Systematic Reviews* (95 articles), *Diabetes, Obesity and Metabolism* (90 articles), *Health and Quality of Life Outcomes* (87 articles), and *BMC Public Health* (81 articles). Together, these 10 journals published 1150 articles, representing 17.77% of the total publications, highlighting the concentration of research output in a few leading journals.

In terms of academic quality, eight of the Top 10 journals belong to the JCR Q1 category, while the remaining two are in Q2 and Q3, respectively. The distribution illustrates the academic significance and clinical relevance of HRQoL research in T2DM, consistently demonstrating high publication quality in these leading journals.

### 3.5. Publication Analysis

Analyzing local citation data provides insights into the foundational and trending topics within the field. Among the 6470 articles included, the Top 10 most locally cited articles were summarized (see Table 5 for details).

Author Ryan J. Anderson published the most locally cited article in *Diabetes Care* in 2001, garnering 280 citations. This study highlighted the significantly increased risk of comorbid depression in patients with T2DM [51]. W. Ken Redekop, who authored the second most cited article in 2002 and published it in *Diabetes Care*, garnered 172 citations. It looked at HRQoL and treatment satisfaction among Dutch people with T2DM, focusing on how obesity and complications affect HRQoL [52]. The third ranked article by P. J. Lustman, published in 2000 in *Diabetes Care*, garnered 164 citations. This study investigated the relationship between depression and glycemic control, revealing a significant association between depression and hyperglycemia in patients with T2DM [53].

Among the Top 10 most locally cited articles, five utilized tools such as EQ-5D and HUI-III to evaluate the health status of patients with T2DM and their relationship with complications. These studies consistently found that complications such as ischemic heart disease, stroke, and neuropathy had significant negative impacts on HRQoL [6, 52, 54–56]. Additionally, three studies focused on the association between depression and other complications in patients with T2DM [51, 53, 57]. One study evaluated the effectiveness of self-management training in improving HRQoL [58], while another analyzed psychosocial issues in patients with T2DM, emphasizing the prevalence and importance of addressing these challenges [59].

Notably, five of the Top 10 most locally cited articles were published in *Diabetes Care*, highlighting its significant academic influence in the field of HRQoL research related to T2DM. These highly cited studies reflect the growing recognition of the impact of complications, depression, and psychosocial issues on HRQoL of patients with T2DM, underscoring the importance of comprehensive interventions in improving their overall health outcomes and well-being.

### 3.6. Keyword and Hotspot Analysis

**Figure 5:**
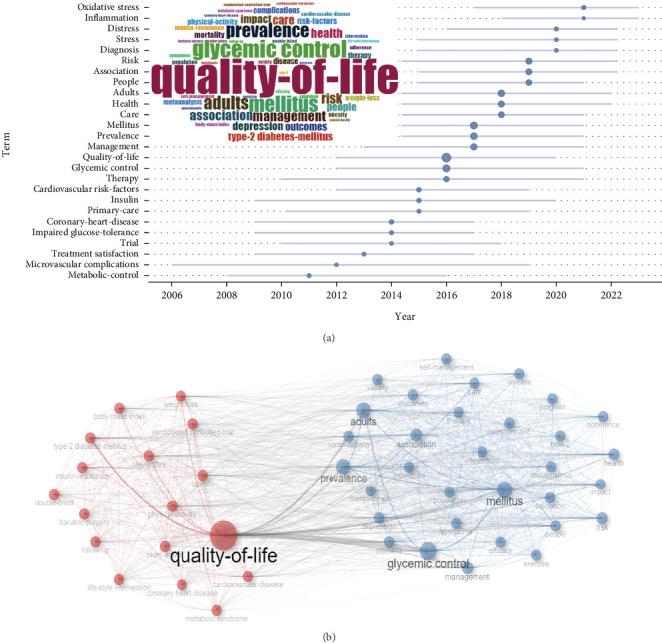
WordCloud, trend topics, and co-occurrence network on HRQoL in patients with T2DM. (a) WordCloud and trend topics on HRQoL in patients with T2DM. (b) Co-occurrence network on HRQoL in patients with T2DM.

Keyword co-occurrence analysis is crucial for identifying key themes in a research field and uncovering their interconnections. In this study, KeyWords Plus from the 6470 included articles were analyzed, resulting in a total of 8233 extracted keywords. We selected the Top 50 most frequently occurring keywords for detailed analysis (see Figure 5 for details).

To better summarize the development and focal areas of HRQoL research in T2DM, we constructed a table highlighting key trends and insights across publication patterns, research themes, and global contributions (see Table 6).

Among these keywords, “quality-of-life” ranked highest, appearing 2043 times, underscoring that HRQoL is the central focus of this research area. “Glycemic control” ranked second with 1026 occurrences, followed by “mellitus” with 944 occurrences. Other frequently appearing keywords included “prevalence” (*n* = 831), “adults” (*n* = 799), “management” (*n* = 621), “risk” (*n* = 606), “association” (*n* = 601), “health” (*n* = 552), “care” (*n* = 539), and “depression” (*n* = 502).

These high-frequency keywords reflect the main themes and research directions within HRQoL studies. “QoL” emerges as the core outcome of HRQoL research, permeating the entire field. Keywords such as “glycemic control,” “association,” and “depression” highlight critical factors influencing HRQoL in patients with T2DM, such as blood glucose management, depression, and associated complications. The prominence of “management” emphasizes the importance of diabetes care and management strategies, reflecting researchers' focus on optimizing patient care and self-management.

The pattern of high-frequency keywords over time showed that early keywords like “microvascular complications,” “metabolic control,” and “treatment satisfaction” showed up more often. This suggests that basic research on metabolic control and clinical complications in T2DM was more important in the early years of the study. From 2014 to 2018, the keywords “glycemic control,” “therapy,” and “management” had a significantly higher increase in frequency, showing a gradual increase in researchers' interest in optimizing glycemic regulation, reducing vascular complications, and improving care strategies. And after 2018, keywords such as “oxidative stress,” “inflammation,” “stress,” and “distress” began to grow rapidly in frequency, suggesting a gradual shift in the direction of research toward exploring diabetes-related biological mechanisms (e.g., oxidative stress and inflammation) as well as mental health issues (e.g., stress and mental burden).

Overall, the way these high-frequency keywords have changed over time suggests that earlier studies were more focused on looking into clinical complications and metabolic control. And studies in recent years have taken a more comprehensive approach, incorporating mental health, biological stressors, and patient management strategies to improve HRQoL in people with T2DM. This change reflects researchers' increasingly comprehensive understanding and also indicates that the breadth and depth of research in this area are expanding.

## 4. Discussion

This study employs bibliometric methods to systematically analyze research on HRQoL in patients with T2DM. It provides a comprehensive overview of the current research landscape, development trends, and key information regarding countries, institutions, authors, and journals. By identifying factors influencing the HRQoL of patients with T2DM, this study is aimed at assisting researchers and healthcare professionals in pinpointing research priorities, fostering collaboration, and devising targeted measures to improve HRQoL in patients with T2DM.

### 4.1. Bibliometric Analysis

The field of HRQoL research for T2DM has witnessed rapid growth. A total of 29,892 authors have contributed to 6470 publications distributed across 1400 journals. Since 2000, the volume of publications has increased significantly, with 40.03% of articles published in the past 5 years (2020–2024), reflecting the field's growing activity and importance. This surge aligns closely with the increasing global burden of T2DM and the healthcare sector's shift toward patient-centered evaluation frameworks. Currently, more than 537 million adults worldwide are living with T2DM, a number projected to rise to 783 million by 2045 [60]. Advances in novel therapies, such as GLP-1 receptor agonists [61] and SGLT2 inhibitors [62], along with digital health technologies like continuous glucose monitoring and telemedicine [63], have enabled better management of complications and extended patient lifespans. More and more, multidimensional assessment systems that look at physical, mental, and social health are replacing traditional clinical evaluation metrics. This is causing HRQoL research to quickly grow.

Globally, 120 countries and regions have contributed to HRQoL research in patients with T2DM. The United States leads the field with 1620 articles and 98,354 citations, reflecting its significant research influence and academic contributions. China ranks second with 622 articles (9.61% of total publications), yet its average citations per article (15.50) lag significantly behind the United Kingdom (62.70), the United States (60.70), and the Netherlands (53.10). This highlights opportunities for improvement in China's research quality and international impact. Among institutions, Harvard University ranks first with 325 publications, followed by the University of California System (258) and the University of Copenhagen (254). Six of the Top 10 contributing institutions are based in the United States, further underscoring its dominance in this field.

The analysis also highlights the imbalance in global research contributions. Among the Top 10 publishing countries, only China is classified as a developing country, while the others are developed nations. Developed countries benefit from advanced healthcare systems, robust research infrastructure, and substantial funding, enabling high-quality clinical and foundational research. Their findings often shape global research standards and foster international collaboration. However, the applicability of findings from developed countries to developing nations is limited due to disparities in healthcare systems, socioeconomic determinants of health, and cultural contexts. Considering that 75% of T2DM patients reside in developing countries—primarily China and India, with 145 million and 74 million patients, respectively [2, 64]—and that urbanization, dietary changes, and limited access to healthcare and education further exacerbate T2DM prevalence in regions such as South Asia, Southeast Asia, South America, and sub-Saharan Africa [4], research priorities should shift geographically. Strengthening collaborations, building research capacity, and formulating culturally and economically tailored health initiatives in low- and middle-income countries will be critical for ensuring equitable and inclusive global research.

Keyword co-occurrence analysis reveals that HRQoL research in T2DM focuses on multidimensional impacts spanning physiological, psychological, and social domains. High-frequency keywords such as “quality of life,” “glycemic control,” and “depression” underscore core research themes, including the effects of complications (e.g., neuropathy and cardiovascular disease) on HRQoL, the role of psychological factors, and the effectiveness of self-management interventions. Early research emphasized clinical complications and metabolic control, as reflected in keywords such as “microvascular complications” and “metabolic control.” From 2014 to 2018, the focus shifted to “glycemic control,” “therapy,” and “management,” indicating increasing interest in optimizing glucose regulation, reducing vascular complications, and improving care strategies. Since 2018, terms like “oxidative stress,” “inflammation,” “stress,” and “distress” have gained prominence, highlighting a transition toward integrating biological mechanisms (e.g., oxidative stress and inflammation) and psychological health into the broader HRQoL framework.

This shift reflects a growing recognition of the multifaceted factors influencing HRQoL. Early studies centered on metabolic and clinical outcomes, while recent research integrates psychological, biological, and patient-centered management strategies to holistically improve HRQoL in T2DM patients.

Meanwhile, the application of digital health technologies, such as wearable devices, telemedicine, and artificial intelligence (AI), is emerging as a research hotspot. These tools enable real-time HRQoL monitoring, personalized interventions, and early identification of high-risk patients. AI-powered predictive analytics and tailored healthcare solutions offer significant potential to enhance patient outcomes while addressing disparities across diverse socioeconomic and cultural settings.

Moreover, researchers are increasingly designing culturally and socioeconomically appropriate care strategies to improve patient autonomy and overall health. Initiatives such as structured education programs (e.g., Diabetes Education and Self-Management for Ongoing and Newly Diagnosed [DESMOND] and Dose Adjustment For Normal Eating [DAFNE]) and psychosocial support are essential for empowering patients and fostering adherence to self-management practices.

### 4.2. Factors Affecting HRQoL in Patients With T2DM

To effectively aid researchers and healthcare professionals in devising targeted measures for improving HRQoL in patients with T2DM, this study examined key influencing factors. Current evidence highlights the critical roles of sleep disturbances [7], depression [6, 65, 66], educational level [2, 5], income [5, 65], gender [6, 65], diabetes duration [2, 3], treatment modalities [2, 6, 65], lifestyle factors [5], microvascular complications [6], heart failure [6], and other diabetes-related complications and comorbidities. Additionally, factors such as age, marital status, obesity, and dietary control remain contentious. Some studies identify age [2, 67], marital status [2], obesity [2, 52], and dietary control [68] as significant determinants of HRQoL, whereas others report no significant associations [3, 6, 69–72].

Among these factors, psychological health, sleep disturbances, and socioeconomic conditions warrant special emphasis due to their profound impact on HRQoL. Sleep disturbances are pervasive in T2DM patients [7, 73–75]. Poor sleep quality or insufficient sleep not only damages physical health but also reduces daily functioning and self-management adherence, triggering a cascade of health problems. These issues significantly impair HRQoL, creating a vicious cycle where fatigue and poor mental states exacerbate psychological burdens [7, 73]. Psychological health issues such as anxiety, depression, and emotional distress are also critical determinants of HRQoL [5, 66]. Psychosocial factors influence health behaviors and treatment adherence through complex mechanisms. Previous studies demonstrate that psychological distress is associated with reduced treatment adherence [76], while depression correlates with poor glycemic control [77] and increased mortality [77]. In contrast, better psychological health is linked to improved glycemic control, higher adherence to healthy behaviors, and lower mortality rates [78]. Increased psychological stress during daily life and treatment often leads to maladaptive coping behaviors, such as neglecting self-care, which, in turn, heightens the risk of complications, further deteriorating both physical and mental health.

Socioeconomic status, including education level and income, also plays a pivotal role in shaping HRQoL. Higher education and income levels enable better access to healthcare resources and information, promoting treatment adherence and satisfaction. In contrast, individuals with lower education levels often lack the knowledge and skills necessary for effective self-management, leading to poorer outcomes. Empowering patients with autonomy in their care has been shown to enhance understanding of their health, improve treatment adherence, and increase satisfaction.

Addressing these factors requires a multifaceted approach that considers clinical, psychological, behavioral, and social dimensions: (1) Clinical interventions: Personalized treatment plans, including advanced therapies such as GLP-1 receptor agonists and SGLT2 inhibitors, can optimize glycemic control, simplify medication regimens, reduce disease burden, and prevent complications. (2) Psychological and educational support: Strengthening psychological health through interventions such as cognitive behavioral therapy (CBT), mindfulness-based programs, and peer support groups can alleviate diabetes-related distress, anxiety, and depression [5, 66]. (3) Behavioral interventions: Structured physical activity programs tailored to patients' abilities, combined with personalized dietary counseling, can enhance physical functioning and promote overall health [3]. (4) Social interventions: Family involvement and culturally tailored education programs, such as DESMOND or DAFNE, can provide a supportive environment while equipping patients with the knowledge and skills needed for effective self-management.

Additionally, digital health technologies offer significant potential to improve HRQoL. Mobile applications and wearable devices can provide real-time feedback on glycemic control and lifestyle behaviors. Telemedicine platforms and AI-powered tools can identify high-risk patients and deliver personalized interventions. By integrating these strategies, healthcare professionals can adopt a patient-centered approach, develop targeted health policies, improve care delivery, and effectively manage T2DM, ultimately enhancing patients' HRQoL.

## 5. Limitations

This study has several inherent limitations related to the bibliometric methods and data sources employed. First, the analysis was based exclusively on the WoSCC, a widely used and authoritative database. However, this single-source approach may have excluded relevant publications indexed in other databases such as Scopus, PubMed, and Embase, potentially limiting the scope and introducing selection bias. Second, the evaluation of research impact relied predominantly on citation counts, which tend to underestimate the influence of recently published studies due to limited accumulation time. Moreover, citation counts can be influenced by self-citation, citation circles, and publication language and may not accurately reflect research quality, innovation, or clinical relevance—especially in emerging or interdisciplinary areas. Third, keyword co-occurrence analysis—while effective in mapping thematic trends—can be biased by author- or journal-supplied keywords, inconsistent terminology, or indexing artifacts. For instance, synonymous terms may fragment results, while underused but conceptually important terms may be underrepresented. This may lead to an incomplete or skewed representation of research themes.

Future studies should consider integrating data from multiple bibliographic sources and employing standardized or ontology-based keyword harmonization. The adoption of complementary metrics such as altmetrics (e.g., online attention, social media mentions, and policy citations) could offer a more balanced and real-time view of research dissemination and influence. Finally, while this bibliometric analysis offers a macrolevel understanding of HRQoL research in T2DM, it is essential to interpret the findings in context. The translation of these insights into clinical and public health policy remains complex. For example, the dominance of research output from high-income countries raises concerns about the applicability of evidence to low- and middle-income settings, where the diabetes burden is greatest. Greater emphasis should be placed on aligning research efforts with real-world clinical needs, resource constraints, and cultural contexts to ensure equitable and practical improvements in patient outcomes.

## 6. Conclusion

This study conducted a systematic bibliometric analysis of the research landscape on HRQoL in patients with T2DM. A total of 29,892 authors from 120 countries or regions have contributed to 6470 publications, distributed across 1400 journals. Notably, the past 5 years have seen the publication of 40.03% of these studies, underscoring the field's growing importance and vitality. The United States leads globally in terms of publication output and research influence.

Across the included studies, several key factors were consistently reported to impact HRQoL in T2DM patients. These include psychological conditions such as depression, anxiety, and diabetes-related emotional distress; sleep disturbances; and diabetes-related complications such as neuropathy, cardiovascular disease, and retinopathy. Socioeconomic status—particularly low income, low education, and limited healthcare access—was frequently associated with reduced HRQoL, especially in developing countries. Lifestyle factors like physical inactivity and poor diet also emerged as recurrent themes. Early HRQoL research primarily emphasized glycemic control and complication management. However, recent studies have adopted a multidimensional and patient-centered perspective, integrating physiological, psychological, social, and behavioral dimensions to more holistically support T2DM patients. Advances in digital health tools, psychosocial support interventions, and culturally sensitive education programs are increasingly being incorporated to improve QoL outcomes.

Future research should focus on building research capacity in developing countries, where the burden of T2DM is disproportionately high. Strengthening international collaborations and developing personalized, culturally tailored interventions can drive global improvements in HRQoL. Such advancements not only promise enhanced QoL for T2DM patients but also provide valuable insights for optimizing global healthcare policies.

## Figures and Tables

**Table 1:** Top 10 countries with corresponding authors of publications on HRQoL in patients with T2DM.

**Country**	**Publications**	**SCP**	**MCP**	**Frequency**	**MCP_Ratio**
United States	1620	1334	286	0.250	0.177
China	622	504	118	0.096	0.190
United Kingdom	529	311	218	0.082	0.412
Australia	356	220	136	0.055	0.382
Italy	258	181	77	0.040	0.298
Netherlands	252	165	87	0.039	0.345
Canada	231	175	56	0.036	0.242
Germany	210	140	70	0.032	0.333
Japan	184	164	20	0.028	0.109
Denmark	154	98	56	0.024	0.364

*Note:* Frequency, number of publications in this country/total number of publications; MCP_Ratio, MCP/publications.

Abbreviations: MCP, multiple country publications; SCP, single country publications.

**Table 2:** Commonly used HRQoL instruments in T2DM studies and their assessed domains.

**Instrument**	**Assessed domains**	**T2DM-relevant physical issues**	**T2DM-relevant psychosocial issues**	**Notes**
SF-36 (Short Form-36)	Physical functioning, role limitations (physical and emotional), vitality, mental health, social functioning, bodily pain, general health perceptions	Fatigue, mobility limitations, pain	Depression, anxiety, emotional distress, reduced social interaction	Widely used generic instrument
EQ-5D (EuroQol-5 Dimensions)	Mobility, self-care, usual activities, pain/discomfort, anxiety/depression	Pain, daily activity limitation	Depression, anxiety	Short, preference-based tool for utility scoring
WHOQOL-BREF	Physical health, psychological health, social relationships, environment	Sleep disturbances, energy, dependence on medication	Body image, negative feelings, social support	Developed by WHO; cross-cultural validity
SF-12	Physical and mental component summary scores	Physical function, role limitations	Mental health, social functioning	Shorter version of SF-36
DQoL (Diabetes Quality of Life)	Satisfaction, impact, worry (social/vocational), worry (diabetes-related)	Treatment burden, symptoms	Fear of complications, emotional burden	Specifically for diabetes populations
ADDQoL (Audit of Diabetes-Dependent Quality of Life)	Impact of diabetes on 19 life domains + overall QoL	Work, diet, mobility	Self-esteem, social life, future concerns	Allows individual weighting of domains
HUI (Health Utilities Index)	Vision, hearing, speech, ambulation, dexterity, emotion, cognition, pain	Sensory impairment, pain, mobility	Cognitive limitation, emotional issues	Suitable for utility-based analysis
PROMIS	Physical, mental, and social health	Pain, physical function	Depression, anxiety, fatigue, social roles	Modular, adaptable, increasingly adopted

**Table 3:** Top 10 productive authors with publications on HRQoL in patients with T2DM.

**Rank**	**Author**	**Current affiliations and countries**	**Number of publications**	**Local citations**
1	Kamlesh Khunti	University of Leicester, United Kingdom	55	328
2	Frans Pouwer	University of Southern Denmark, Denmark	40	277
3	Melanie J Davies	University of Leicester, United Kingdom	39	232
4	F. J. Snoek	Departments of Amsterdam Public Health Research Institute, Netherlands	37	478
5	Guy E H M Rutten	University Medical Centre Utrecht, Netherlands	35	339
6	E. Egede	University at Buffalo, United States	30	148
7	Ping Zhang	Centers for Disease Control and Prevention, United States	30	223
8	Kristina Secnik Boye	Eli Lilly and Company, United States	29	205
9	Antonio Nicolucci	Center for Outcomes Research and Clinical Epidemiology, Italy	29	251
10	Jeffrey A. Johnson	University of Alberta, Canada	27	192

**Table 4:** Top 10 journals by number of publications on HRQoL in patients with T2DM.

**Rank**	**Journal**	**Publications (%)** ^**a**^	**CumPub (%)** ^**b**^	**IF (2023)** ^**c**^	**JCR category (SCIE)**
1	*Diabetes Research and Clinical Practice*	168 (2.60%)	168 (2.60%)	6.1, Q1	Endocrinology and metabolism
2	*Diabetic Medicine*	164 (2.53%)	332 (5.13%)	3.2, Q2	Endocrinology and metabolism
3	*Diabetes Care*	149 (2.30%)	481 (7.43%)	14.8, Q1	Endocrinology and metabolism
4	*BMJ Open*	115 (1.78%)	596 (9.21%)	2.4, Q1	Medicine, general, and internal
5	*PLOS One*	102 (1.58%)	698 (10.79%)	2.9, Q1	Multidisciplinary sciences
6	*Diabetes Therapy*	99 (1.53%)	797 (12.32%)	2.8, Q3	Endocrinology and metabolism
7	*Cochrane Database of Systematic Reviews*	95 (1.47%)	892 (13.79%)	8.8, Q1	Medicine, general, and internal
8	*Diabetes, Obesity and Metabolism*	90 (1.39%)	982 (15.18%)	5.4, Q1	Endocrinology and metabolism
9	*Health and Quality of Life Outcomes*	87 (1.34%)	1069 (16.52%)	3.2, Q1	Health care sciences and services
10	*BMC Public Health*	81 (1.25%)	1150 (17.77%)	3.5, Q1	Public, environmental, and occupational health

Abbreviation: SCIE, Science Citation Index Expanded.

^a^Percentage of total published articles in the journal.

^b^Cumulative publications as a percentage of total publications.

^c^Journal impact factor (Journal Citation Reports 2023).

**Table 5:** Top 10 most locally cited publications on HRQoL in patients with T2DM.

**Rank**	**Title**	**First author**	**Year**	**Journal**	**Local citations**
1	The Prevalence of Comorbid Depression in Adults With Diabetes: A Meta-Analysis	Ryan J. Anderson	2001	*Diabetes Care*	280
2	Health-Related Quality of Life and Treatment Satisfaction in Dutch Patients With Type 2 Diabetes	W. Ken Redekop	2002	*Diabetes Care*	172
3	Depression and Poor Glycemic Control: A Meta-Analytic Review of the Literature	P J Lustman	2000	*Diabetes Care*	164
4	Association of Depression and Diabetes Complications: A Meta-Analysis	M De Groot	2001	*Psychosomatic Medicine*	157
5	Estimating Utility Values for Health States of Type 2 Diabetic Patients Using the EQ-5D (UKPDS 62)	P Clarke	2002	*Medical Decision Making*	119
6	Effectiveness of Self-Management Training in Type 2 Diabetes: A Systematic Review of Randomized Controlled Trials	Susan L. Norris	2001	*Diabetes Care*	116
7	Health-Related Quality of Life in Diabetes: The Associations of Complications With EQ-5D Scores	Oddvar Solli	2010	*Health and Quality of Life Outcomes*	92
8	Valuing Health-Related Quality of Life in Diabetes	J. Todd Coffey	2002	*Diabetes Care*	90
9	Correlates of Health-Related Quality of Life in Type 2 Diabetes	D. J. Wexler	2006	*Diabetologia*	88
10	Psychosocial Problems and Barriers to Improved Diabetes Management: Results of the Cross-National Diabetes Attitudes, Wishes and Needs (DAWN) Study	M. Peyrot	2005	*Diabetic Medicine*	86

**Table 6:** Summary of key trends and insights in HRQoL research on Type 2 diabetes mellitus (T2DM).

**Dimension**	**Key findings/highlights**
Publication trends	– 6470 articles published by 29,892 authors across 1400 journals – 40.03% published between 2020 and 2024, showing rapid recent growth
Global burden	– > 537 million adults living with T2DM globally; projected to reach 783 million by 2045
Research hotspots	– Shift from metabolic/clinical focus to multidimensional assessment (physical, mental, social) – Rise in keywords: “quality of life,” “depression,” “self-management,” “stress”
Top contributing countries	– United States: 1620 articles, 98,354 citations – China: 622 articles, lower average citations (15.50) – United Kingdom, Netherlands: higher citation impact (62.70, 53.10)
Leading institutions	– Harvard University (325), University of California System (258), University of Copenhagen (254) – 6 of the Top 10 institutions are in the United States
Research inequality	– Among the Top 10 countries, only China is a developing nation – 75% of T2DM patients live in developing regions (esp. China, India, South Asia, and Africa)
Emerging technologies	– Use of AI, wearable devices, and telemedicine for HRQoL monitoring and personalized care
Key HRQoL themes	– Physical: neuropathy, retinopathy, fatigue, mobility limitations – Psychological: depression, anxiety, diabetes distress – Social: self-perception, social isolation
Common interventions	– GLP-1RAs, SGLT2i, continuous glucose monitoring – Structured education (e.g., DESMOND and DAFNE), psychosocial support programs
Measurement tools	– SF-36, EQ-5D, WHOQOL, SF-12, DQoL, ADDQoL, HUI, PROMIS
Future directions	– Strengthening research capacity in LMICs – Designing culturally tailored and cost-effective strategies – Promoting interdisciplinary and collaborative HRQoL research

## Data Availability

The data and code supporting the findings of this study are available from the corresponding author upon reasonable request.
